# Transparent Electrothermal Heaters Based on Vertically-Oriented Graphene Glass Hybrid Materials

**DOI:** 10.3390/nano9040558

**Published:** 2019-04-06

**Authors:** Lingzhi Cui, Kejian Cui, Haina Ci, Kaiqiang Zheng, Huanhuan Xie, Xuan Gao, Yanfeng Zhang, Zhongfan Liu

**Affiliations:** 1 Center for Nanochemistry (CNC), Beijing National Laboratory for Molecular Sciences, College of Chemistry and Molecular Engineering, Peking University, Beijing 100871, China; cuilz-cnc@pku.edu.cn (L.C.); cihn-cnc@pku.edu.cn (H.C.); xiehh-cnc@pku.edu.cn (H.X.); 2 Beijing Graphene Institute, Beijing 100091, China; cuikj@bgi-graphene.com (K.C.); zhengkq@bgi-graphene.com (K.Z.); gaoxuan@bgi-graphene.com (X.G.); 3 Academy for Advanced Interdisciplinary Studies, Peking University, Beijing 100871, China; 4 Department of Materials Science and Engineering, College of Engineering, Peking University, Beijing 100871, China

**Keywords:** graphene, transparent heater, PECVD

## Abstract

Transparent heating devices are widely used in daily life-related applications that can be achieved by various heating materials with suitable resistances. Herein, high-performance vertically-oriented graphene (VG) films are directly grown on soda-lime glass by a radio-frequency (rf) plasma-enhanced chemical vapor deposition (PECVD) method, giving reasonable resistances for electrothermal heating. The optical and electrical properties of VG films are found to be tunable by optimizing the growth parameters such as growth time, carrier gas flow, etc. The electrothermal performances of the derived materials with different resistances are thus studied systematically. Specifically, the VG film on glass with a transmittance of ~73% at 550 nm and a sheet resistance of ~3.9 KΩ/□ is fabricated into a heating device, presenting a saturated temperature up to 55 °C by applying 80 V for 3 min. The VG film on the glass at a transmittance of ~43% and a sheet resistance of 0.76 KΩ/□ exhibits a highly steady temperature increase up to ~108 °C with a maximum heating rate of ~2.6 °C/s under a voltage of 60 V. Briefly, the tunable sheet resistance, good adhesion of VG to the growth substrate, relative high heating efficiency, and large heating temperature range make VG films on glass decent candidates for electrothermal related applications in defrosting and defogging devices.

## 1. Introduction

Electrically driven transparent heaters have attracted a lot of attention in broad areas containing defogging in automobiles, outdoor displays, heating retaining windows, and other heating systems [1]. To date, the customarily employed materials like strips of metal alloys (such as Fe-Cr-Al or Ni-Cr) are often used in commercial heaters. However, they present several drawbacks like a heavy weight, opacity, and low heating efficiency [2,3]. Therefore, the conductive metal-oxide represented by indium tin oxide (ITO) has been commercially used for fabricating conventional transparent heaters for its extra-high transparency and conductivity [4]. However, disadvantages lie in its intolerance to acids or bases, fragile under bending deformation, as well as the limited reserves of indium, etc. Consequently, the use of many alternative materials have been attempted to serve as heating media. Metal nanowires such as silver nanowires have been developed to be used in the stretchable, wearable transparent heaters [5], while facing the challenge of easy oxidation in the atmosphere. Carbon nanotubes (CNTs) also possess impressive electrical, optical, and thermal properties, and the related heating device was first fabricated by Han and co-workers with the achievement of a remarkable heating performance [6]. However, drawbacks lie in the multi-step process and the necessity to use harsh chemicals, since CNTs need to be purified, dispersed, sonicated and transferred onto targeted substrates. 

Graphene, a two-dimensional layered material which possesses sp^2^-hybridized carbons, has attracted a lot of attention since it was first obtained in 2004 [7]. The excellent electrical [8], optical [9], mechanical [10] and thermal conductive [11] properties endow graphene with versatile applications in varied fields [12], including sensors [13], transparent electrodes [14,15], field-effect transistors [16] and so on. Up to now, various methods have been developed to achieve graphene, such as mechanical exfoliation [7], reduction of graphene oxide [15], anodic bonding [17], epitaxial growth [18] and chemical vapor deposition [19]. Graphene films assembled by chemically reduced graphene oxides (RGO) were firstly used to fabricate transparent heating devices and displayed decent performances [20]. However, the treatment of reduced graphene (RGO) needs high-temperature annealing over 800 °C. Transfer of graphene on metals onto targeting substrates is another promising method to fabricate transparent heating devices [21,22,23]. But it always requires a complicated transfer process, which customarily introduces breakage, wrinkle, and contaminations in the derived graphene. Alternatively, vertically-oriented graphene (VG) has many unique properties, containing free-standing, non-agglomerated morphology, a large amount of exposed edges, etc. [24,25,26]. More importantly, the extremely high in-plane conductivity of graphene can be effectively used, avoiding the sheet-to-sheet resistance. The VG films are thus proposed to serve as perfect heating media. In particular, the combination of VG films and traditional functional substrates like glass should be more promising for related applications. 

Herein, VG films were directly grown on soda-lime glass by using a low temperature (~600 °C) radio frequency (rf) plasma-enhanced chemical vapor deposition (PECVD) route using CH_4_ as the precursor. The optical and electrical properties of VG films were precisely controlled by alternating the growth parameters, especially the growth time. Accordingly, the resulted VG films are featured with a broad range of tunable transparency levels at 20–80% and sheet resistance levels of 250–5600 Ω·sq^−1^. The as-grown VG films on glass hybrids were then manufactured to transparent heating templates for the functions of defogging and defrosting, showing relatively high heating efficiency, uniform temperature distribution, low electrical consumption and a wide range of heating temperature. The highly tunable range of sheet resistance, high chemical stability and good adhesion of graphene to the growth substrates were considered to be the unique properties of the current system, compared with other materials such as metal alloys related or RGO-related heating media in the electrothermal devices.

## 2. Materials and Methods

### 2.1. Synthesis of Vertically-Oriented Graphene

Uniform VG films were directly grown on soda-lime glass by an rf-PECVD method. In a typical PECVD process, the used soda-lime glass substrate was cleaned with deionized water, ethanol and acetone before loading into the quartz tube placed inside a three-zone furnace. CH_4_ was selected as the carbon source. A copper coil was used to assist the generation of plasma, which was placed ~40 cm away from the glass substrate. A vacuum pump was used to keep the system at low pressure (~15 Pa). The typical growth temperature was set at ~600 °C. The thicknesses of the VG samples were controlled by varying the growth time, plasma generating power, flow rate of the precursor gas, and growth temperature. After the optimization of the other parameters at a steady condition, the growth time is selected as the fundamental parameter.

### 2.2. Characterizations

The samples were systematically characterized using Raman spectroscopy (LabRAM HR Evolution, Horiba, Paris, France; 532 nm laser excitation, 100× objective lens), scanning electron microscopy (SEM, FEI Quattro S, IBM, Waltham, MA, USA, operating at 1 kV), four-probe resistance measurement meter (RTS-4, Guangzhou 4-probe Tech Co. Ltd., Guangzhou, China), atomic force microscopy (AFM, BRUKER Dimension Icon, Karlsruhe, Germany), infrared imaging system (FLIR A615, Täby, Sweden) and UV-Vis spectroscopy (Perkin-Elmer Lambda 950 spectrophotometer, Waltham, MA, USA).

## 3. Results

### 3.1. Structural Characterizations of Vertically-Oriented Graphene Films

**Figure 1:**
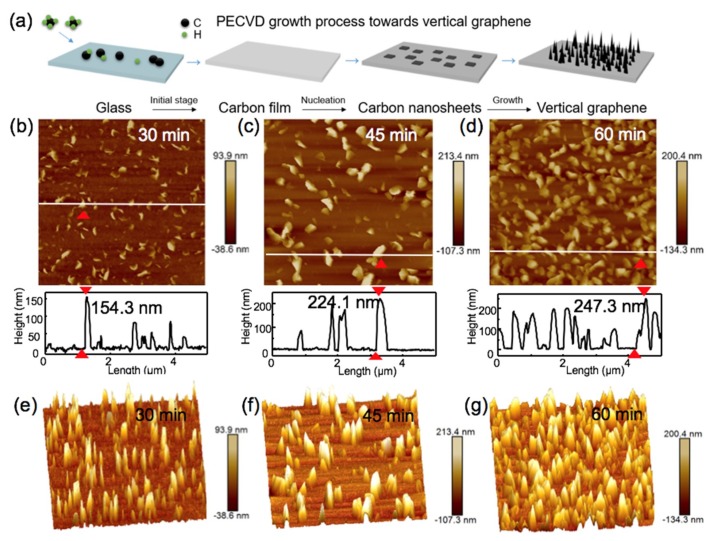
Plasma-enhanced chemical vapor deposition (PECVD) growth towards the formation of vertically-oriented graphene (VG). (**a**) Schematic illustration of VG growth by the radio-frequency PECVD (rf-PECVD) method. (**b**–**d**) Typical 2D atomic force microscopy (AFM) height images (5 µm × 5 µm) of directly-grown VG films on soda-lime glass at various growth time: (**b**) 30 min, (**c**) 45 min, (**d**) 60 min, respectively, with the identical growth conditions (10 standard cubic centimeter per minute (sccm) CH_4_, 600 °C, 300 W). Insets show the section view of the images along the white-dashed line direction. The corresponding 3D AFM height images are shown in (**e**–**g**).

The growth mechanism of VG on the soda-lime glass by the rf-PECVD method is summarized in Figure 1a. In the beginning, the carbon source CH_4_ is disassociated into highly reactive carbon species (CH*_x_*, C_2_H*_y_*, and atomic C and H) by the radio-frequency discharge. These active species are first adsorbed onto the glass surface at the temperature of ~600 °C (right below the softening point of soda-lime glass). The irregular cracks, impurities and dangling bonds on the substrate surface serve as nucleation sites, inducing the formation of a horizontal carbon buffer layer. The carbon species then migrate on the buffer layer and form carbon nanosheets or islands with open edges with the increasing growth time, under the effects of localized electric field and the internal stress. After that, carbon nanosheets grow vertically, and the growth is terminated until the closure of open edges, which is determined by the competition between the etching effect in the plasma and the materials deposition [27].

To verify the generation of VG, various comparative experiments with varying growth time were carried out under the identical PECVD growth condition (10 standard cubic centimeter per minute (sccm) CH_4_, 300 W, 600 °C, 15 Pa). The surface morphologies of the VG films deposited on soda-lime glass were first characterized by atomic force microscopy (AFM). Typical 2D and 3D AFM images of VG films on soda-lime glass with different growth times of 30, 45 and 60 min are shown in Figure 1b–g. The height profiles of these samples are plotted along the lines in Figure 1b–d. The red arrows in each image mark the ridge differences. At the initial growth stage (within the early 30 min), a polycrystalline carbon layer was formed on the glass surface Figure 1b,e, and more detailed images are shown in Figure S1. Individual graphene nanosheets of ~154.3 nm high also appeared on the carbon layer. With increasing growth time, this vertical growth was then facilitated by the strain energy at the curved edges, and the nearby VG domains merged with each other and reached ~224 nm at ~45 min (Figure 1c,f). After 60 mins’ growth, the corrugation of the VG nanosheets reached ~247 nm and the density of nanosheets increased gradually, as shown in Figure 1d,g.

The corresponding growth-time-dependent SEM images are also presented in Figure S2. In the early stage of growth (20 min), a polycrystalline carbon buffer layer was formed on the glass surface, accompanied by the formation of a few nanosheets oriented perpendicularly to the glass surface (Figure S2a). As the growth progressed, nucleation sites initialize on the boundaries and dangling bonds induced by ion bombardment, leading to the growth of vertically-oriented graphene (Figure S2b,c). With the increase of growth time to 60 min, the randomly distributed vertical graphene nanosheets cover the whole surface and keep growing (Figure S2d). When the growth time reached 90 min, much larger graphene nanosheets formed with the evolution of network structures (Figure S2e). The cross-section image suggests that these vertically-oriented graphene sheets are around 1 μm high (Figure S2f).

The preference of graphene to grow vertically rather than parallel to the substrate surface can be explained as follows: (1) the irregular cracks and dangling bonds on the substrate surface serve as nucleation sites, and a buffer layer is formed [28]; (2) the edges of buffer layer or defects curve upward, and vertical growth of graphene happens in these curved edges [27]; (3) the diffusion of carbon cations along the vertical nanowalls is enhanced by the electric field in the sheath layer in plasma, as well as by the stronger localized electric field due to the sharp features of VG edges [29,30,31].

**Figure 2:**
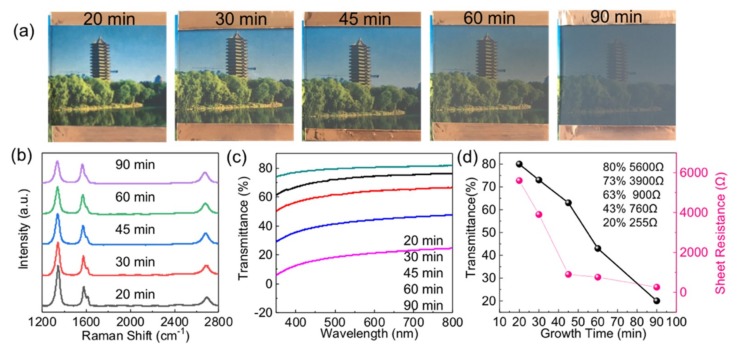
(**a**) Photographs of 5 cm × 5 cm PECVD-derived VG films on the soda-lime glass at 600 °C with varying the growth time from 20, 30, 45, 60 to 90 min, respectively. (**b**) Corresponding Raman spectra of the VG nanosheets films, respectively. (**c**) Corresponding transmittance spectra in the wavelength range of 300–800 nm for VG films on glass, respectively. (**d**) Corresponding sheet resistances and UV-vis transmittance spectra at 550 nm for the VG nanosheets on the glass, respectively.

Figure 2a shows the photographs of the VG films on soda-lime glass samples for 20, 30, 45, 60 and 90 min at 600 °C under the system pressure of 15 Pa. Copper tapes were attached to both sides of the glass for fabricating transparent heating devices, as displayed in the latter part of the work (Figure S3). These samples are marked as 20#, 30#, 45#, 60#, 90#, according to their different growth time. Evidently, the grey contrast of the VG films on glass samples (superimposed on a scenic photo) from left to right increases, which is caused by the increase of the density of VG nanosheets and the lateral expansion of nanosheets. This observation is also supported by the corresponding Raman and transmittance measurements (Figure 2b,c). Raman spectra were also performed on the above mentioned VG nanosheets directly grown on glass. All the spectra are standardized by G peak intensity for comparison. The D, G, and 2D peaks are fitted with the Lorentzian function and the D′ peak fitted by a Fano line shape. Here, the heights of specific peaks are used to indicate the peaks intensities, which are denoted as *I*_D_, *I*_G_, *I*_D′_, *I*_2D_ for the D, G, D′, and 2D peaks, respectively.

The *I*_2D_/*I*_G_ intensity ratio increases from ~0.47 to 0.94 with increasing growth time from 20 to 90 min, as clearly shown in Figure S4a. In addition, the I_D_/I_G_ intensity ratio decreases from 2.1 to 1.2 (Figure S4b), demonstrating the improvement of crystal quality and the expansion of lateral length of the VG nanosheets with an increasing growth time from 20 to 90 min. This can be explained by the empirical equation proposed by Cancado et al. [32], which describes the relation among the in-plane *sp*^2^ crystallite size *L*_a_, the excitation energy of laser source *E*_L_, and *I*_D_/*I*_G_:

$$L_{a}\left(\mathrm{nm}\right)=560/E_{L}^{4}{\left(I_{D}/I_{G}\right)}^{-1}$$

Moreover, D’ band appears around 1610 cm^−1^, corresponding to the highest frequency feature in the density of states [33]. The intensity ratios of *I*_D_/*I*_D’_ were ~5.49, 4.33 and 6.58 at the first growth stage for 20, 30, 45 min, respectively, which indicates the occurrence of obvious vacancy defects [34]. After 45 min of growth, the intensity ratios of *I*_D_/*I*_D’_ were estimated to be ~2.89 and 2.73, suggesting the evolution of abundant grain boundary defects [34,35]. The details are shown in Table S1. Specifically, the transparencies of the VG films on glass (at 550 nm) are tunable from 80%, 73%, 63%, and 43% to 20%, by varying the growth time of VG from 20, 30, 45, and 60 to 90 min, respectively (Figure 2c). The sheet resistance also varies significantly with the values of ~5.60, 3.90, 0.90, 0.76 and 0.26 kΩ/□, accordingly, as shown in Figure 2d. Briefly, this series of results show the wide tunability of the optical and electrical properties of the synthesized VG films on soda-lime glass.

### 3.2. Heating Performances of VG-Based Transparent Heater Devices

**Figure 3:**
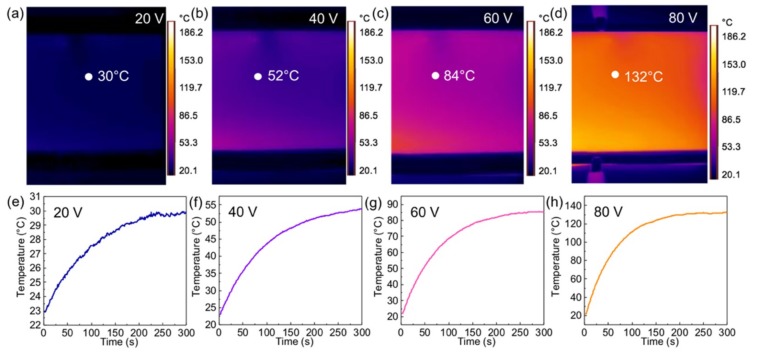
The infrared thermal images of 45# VG films on glass (5 cm × 5 cm, 63%, 900 Ω/□) under different applied voltages, under (**a**) 20 V, (**b**) 40 V, (**c**) 60 V and (**d**) 80 V, respectively. Time versus temperature profiles of the samples with respect to different applied voltages are listed in (**e**) 20 V, (**f**) 40 V, (**g**) 60 V and (**h**) 80 V, respectively.

The unique advantages of the VG nanosheets on soda-lime glass, including the one-step growth strategy, comparatively low growth temperature, reduced sheet resistance, excellent uniformity of transmittance and sheet resistance, and more importantly, its combination with soda-lime glass, make it readily practical for the fabrication of transparent heating devices. Herein, sample 45# (5 cm × 5 cm, 63%, 900 Ω/□) was fabricated into a heater, as it has relatively high transparency and low sheet resistance. Sheet resistance mapping of sample 45# was first performed to address the excellent uniformity (Figure S5). Figure 3 indicates the infrared images of the VG film of 45# by applying different voltages (20, 40, 60, 80 V), respectively. As expected, the final equilibrium temperature increases obviously as the input voltage goes up. And the time consumed to reach the equilibrium temperature is shorter for the sample at higher input voltage. Although there is no inter-digital electrode loaded on the graphene film, obvious heat radiation can be observed at a low input voltage of 20 V. Overall, the temperature distribution is rather homogeneous according to the infrared thermal images showing a uniform color contrast. This is attributed to the uniform sheet resistance/electrical conductivity, as well as the excellent thermal conductivity of the as-grown VG films on glass. It should also be noticed that the size of VG on the glass sample is only limited by the size of the furnace, and it can be enlarged to several inch scales by a large furnace or a different growth route [25]. Therefore, the VG films on glass hybrids are desirable for a wide range of applications in electrothermal heating related aspects.

**Figure 4:**
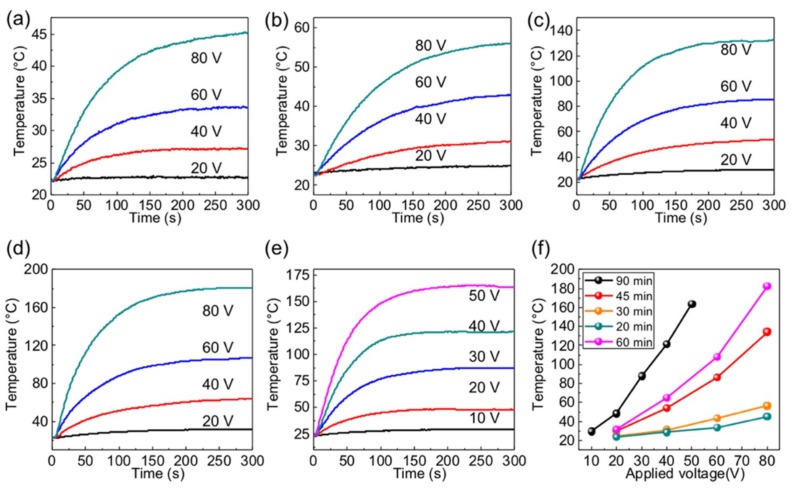
Electrothermal performances of VG films on glass (5 cm × 5 cm) under varied growth times from 20, 30, 45, and 60 min to 90 min, respectively. (**a**–**e**) Time versus temperature profiles under different applied voltages. (**f**) Steady-state temperature versus the applied voltage plots for the different samples.

The as-obtained samples were then fabricated into heaters for electrothermal performance tests. Copper tapes were attached to both sides of the glass as electrodes. An alternating current (ac) was applied to the VG films on soda-lime glass platforms (valid heating area: 5 × 5 cm^2^). Their electrothermal performances were studied under atmospheric conditions, as demonstrated in Figure 4. Upon connecting electrical power, the surface temperature monotonically rises over time until a stable temperature is obtained. With increasing the applied voltage, the steady temperature of a given film surges. Moreover, the longer the growth time (with respect to lowered resistance), the higher the steady temperature was attained at a given voltage. Among these, the VG film obtained by 20 min (20#, 80%, 5600 Ω/□) has a relatively higher sheet resistance, which shows the lowest steady temperature and the lowest heating rate given the same applied voltage.

This can be explained by the relative low transition efficiency from electrical energy to Joule-heating for the current sample. Since *P* = *U*^2^/*R*, where *P* is power, *U* is the applied voltage, *R* is the resistance. Sample 20# (80%, 5600 Ω/□) has a relatively higher sheet resistance, which shows the lowest steady temperature (~32 °C) and the lowest heating rate (0.23 °C/s) given the same applied voltage (60 V) (Figure 3a and the Supporting Information (SI), Figure S6). Therefore, the sample marked with 20# can be used as transparent heating device considering its low electricity consumption (e.g., maximum input power density less than 0.045 W cm^−2^) and good transparency ~80% over the whole vision region. The input power density is lower than that of the RGO-based transparent heater at the same sheet resistance, but with approximately equal transparency under the same applied voltage [15]. In comparison, as shown in Figure 4c–e, the 60# and 90# samples (43%, 760 Ω/□; 20%, 255 Ω/□) show higher steady temperature and heating rates, due to their lower sheet resistances with regard to that of the 45# sample (63%, 900 Ω/□).

Notably, all of the samples can reach the maximum temperature in less than 3 min, highly indicating their rather high electrothermal heating performances. Specifically, the 45# sample (43%, 760 Ω/□) can reach a maximum temperature of ~45 °C under 40 V, and ~130 °C under 80 V. With regard to the 90# sample, the steady temperature can reach ~160 °C under 50 V, exhibiting the maximum heating rates of ~2.6 °C/s at an applied voltage of 40 V (Figure S7). 50 V is the cut off voltage for 90# sample as the temperature can reach over 200 °C when 80 V is applied, which causes copper tape oxidization. Compared to 20# (80%, 5600 Ω/□), the 30# (73%, 3900 Ω/□), 45# (63%, 900 Ω/□), 60# (43%, 760 Ω/□), 90# (20%, 255 Ω/□) samples can achieve higher input power at the same applied voltage due to their improved electrical conductivities. But the maximum input power density is still less than 0.4 W cm^−2^ (see Figure S8). Under the same input power density ~0.4 W cm^−2^, the VG-based heating device can obtain a relatively high heating temperature (~160 °C), twice as high as that of the RGO-based heating devices (~80 °C) [15]. For 45#, 60#, 90# samples, improved electrothermal performances can be realized, while their transparencies were reduced dramatically. Due to their different electrothermal properties, the applications of the hybrid materials can be very different. The performances of the reported electrothermal heaters are summarized in Table S2. Notably, under the same power density, the max heating rate achieved in this work is comparable, or even larger than that reported previously. As for the low transparency films, they can be used as heating devices which do not require high transparency, such as antifogging mirrors in bathrooms and vehicles.

**Figure 5:**
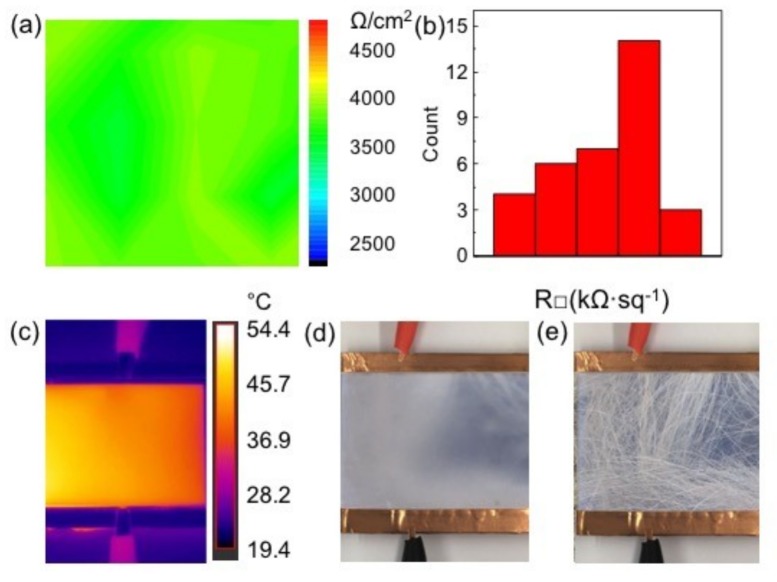
(**a**) Spatial distribution of the sheet resistance of the 30# sample (transmittance ~73%). The map is composed of 36 points, collected from 5 cm × 5 cm graphene glass. (**b**) The statistical distribution of the sheet resistance. (**c**) Infrared temperature image on the VG glass (5 cm × 5 cm) fabricated by the rf-PECVD route. Defrosting results (**d**) before, and (**e**) after heating the 30# VG film by an applied voltage of 40 V.

There are also some applications requiring high transparency and excellent heating performance, such as transparent heaters on building windows or cars. The 20# (80%, 5900 Ω/□) and 30# (73%, 3900 Ω/□) samples with high transparency can be candidates for the above application scenarios. VG has good uniformity at both high and low sheet resistances (30#), as demonstrated in Figure 5a (size: 5 cm × 5 cm, transmittance: ~73%, sheet resistance: ~3900 Ω/□). Detailed statistical distributions of the sample are shown in Figure 5b. With 40 V input voltage, the VG glass shows a uniform temperature distribution of 39.0 ± 1.0 °C, as shown in the infrared thermal image in Figure 5c. It is known that one of the probable applications of the heating performance for VG glass is the defroster device. Herein, the sample 30# (VG films on glass: 73%, 3900 Ω/□) was fabricated into a defroster device in Figure 5d,e, which possessed a good defrosting performance with a completion time of ~120 s under 40 V. In general, the VG directly grown on soda-lime glass by the rf-PECVD method possesses excellent graphene thickness uniformity, high transparency, homogeneous sheet resistance, thus affording advanced defrosting potential in outdoor displays or other equipment requiring transparent, conductive heating films for defogging or temperature maintenance.

## 4. Conclusions

We report the direct synthesis of high-performance VG films on soda-lime glass by a rf-PECVD method. Considering the tunable transparency and resistance, such directly grown VG films on soda-lime glass are exploited to fabricate electrical thermal heating devices. The electrothermal performance was studied in terms of applied voltage and heating rate. Moreover, prototype devices for defogging or defrosting were also fabricated by these VG films on glass hybrids, which possess homogeneous sheet resistance, low electrical consumption, and excellent heating efficiency. In essence, this work provides a reliable method for the production of VG on the glass, as well as presenting a visual demonstration of its applications in electrothermal related fields, especially in traffic transportation, home appliances, or any other industrial fields.

## Supplementary Materials

The following are available online at https://www.mdpi.com/2079-4991/9/4/558/s1, Figure S1: Detailed AFM image of VG film obtained in 30 mins (1.6 μm × 1.6 μm), Figure S2: (a–e) SEM images of graphene nanowalls directly grown on the soda-lime glass for growth periods of 20, 30, 45, 60 and 90 min, respectively, with plasma power 300 W at 600 °C. (f) Cross section SEM image of graphene nanowalls through 90 min growth, Figure S3: Schematically illustration of the structure of the heating devices, Figure S4: (a,b) *I*_2D_/*I*_G_ and *I*_D_/*I*_G_ ratio of different growth time, Figure S5: Sheet resistance mapping of sample 45# indicates the excellent uniformity, Figure S6: Heating rates of (a) 20#, (b) 30#, (c) 45#, (d) 60# at 60 V, Figure S7: Heating rates of (a) 20#, (b) 30#, (c) 45#, (d) 60#, (e) 90# at 40 V, Figure S8: (a) steady-state temperatures vs input power; (b) steady-state temperature vs power density. Table S1: The intensity ratios of *I*_D_/*I*_G_, *I*_D’_/*I*_G_, *I*_D_/*I*_D’_ for growth time of 20, 30, 45, 60 and 90 min, Table S2: Heaters fabricated by carbon-based films and their performances.
