# Supplementary material for: Transparent Electrothermal Heaters Based on Vertically-Oriented Graphene Glass Hybrid Materials
Source: Nanomaterials (Basel). 2019 Apr 6;9(4):558. doi: 10.3390/nano9040558 (PMC6523228; doi:10.3390/nano9040558)
Supplement: Supplementary file 1 [file nanomaterials-09-00558-s001.pdf]

Supplementary Materials

Transparent Electrothermal Heaters Based on Vertically-Oriented Graphene Glass Hybrid Materials

Lingzhi Cui [1,2], Kejian Cui [2], Haina Ci [1,2,3], Kaiqiang Zheng [2], Huanhuan Xie [1,2], Xuan Gao [2], Yanfeng Zhang [1,2,4,*] and Zhongfan Liu [1,2,*]

1 Center for Nanochemistry (CNC), Beijing National Laboratory for Molecular Sciences, College of Chemistry and Molecular Engineering, Peking University, Beijing 100871, China; cuilz-cnc@pku.edu.cn (L.C.); cihn-cnc@pku.edu.cn (H.C.); xiehh-cnc@pku.edu.cn (H.X.)

2 Beijing Graphene Institute, Beijing 100091, China; cuikj@bgi-graphene.com (K.C.); zhengkq@bgi-graphene.com (K.Z.); gaoxuan@bgi-graphene.com (X.G.)

3 Academy for Advanced Interdisciplinary Studies, Peking University, Beijing 100871, China

4 Department of Materials Science and Engineering, College of Engineering, Peking University, Beijing 100871, China

* Correspondence: yanfengzhang@pku.edu.cn (Y.Z.); zfliu@pku.edu.cn (Z.L.); Tel.: +86-010-62767065 (Y.Z.); +86-010-62757157 (Z.L.)

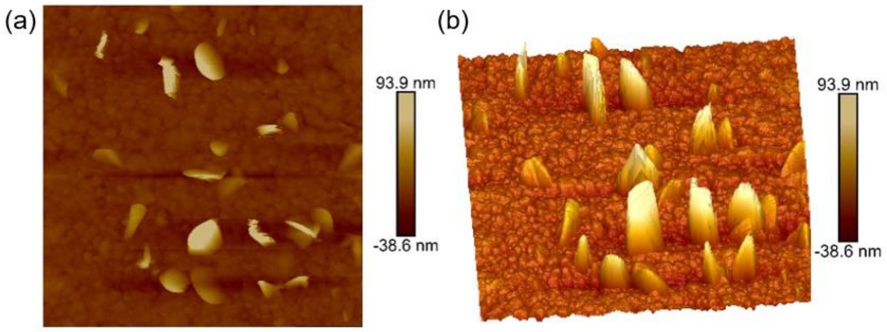

Figure S1. Detailed AFM image of VG film obtained in 30min (1.6 μm × 1.6 μm).

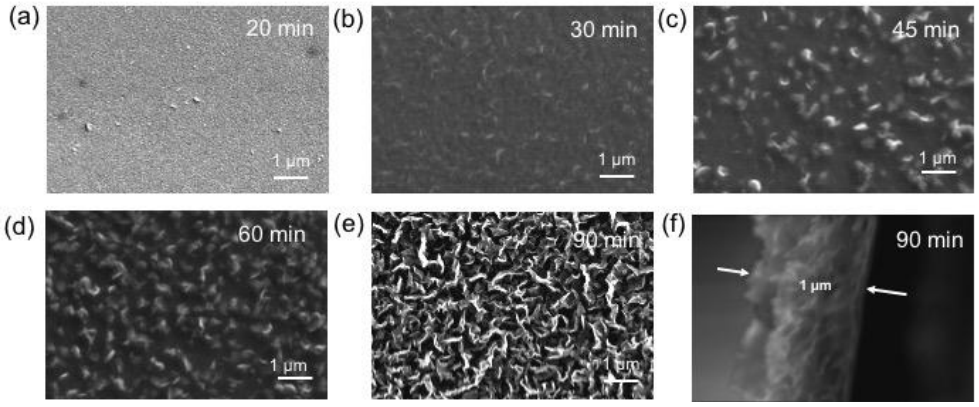

Figure S2. (**a–e**) SEM images of graphene nanowalls directly grown on the soda-lime glass for growth periods of 20, 30, 45, 60 and 90 min, respectively, with plasma power 300 W at 600 °C. (**f**) Cross section SEM image of graphene nanowalls through 90 min growth.

Table S1. The intensity ratios of I_D/I_G, $I_{D'}/I_G$, $I_D/I_{D'}$ for growth time of 20, 30, 45, 60 and 90 min.

Growth time	I_D/I_G	$I_{D'}/I_G$	$I_D/I_{D'}$
20min	2.12	0.39	5.49
30min	1.72	0.40	4.33
45min	1.62	0.25	6.58
60min	1.42	0.49	2.89
90min	1.16	0.42	2.73

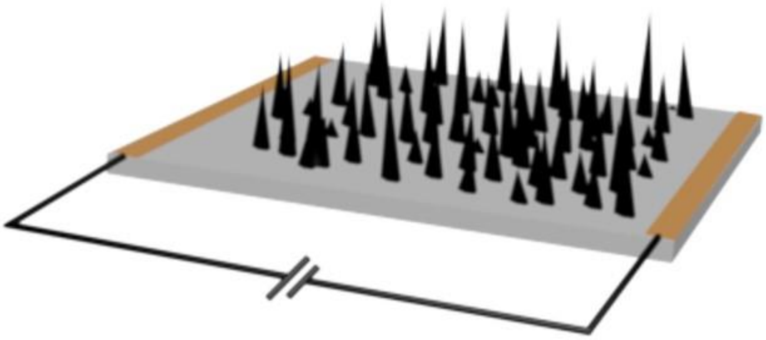

Figure S3. Schematically illustration of the structure of the heating devices.

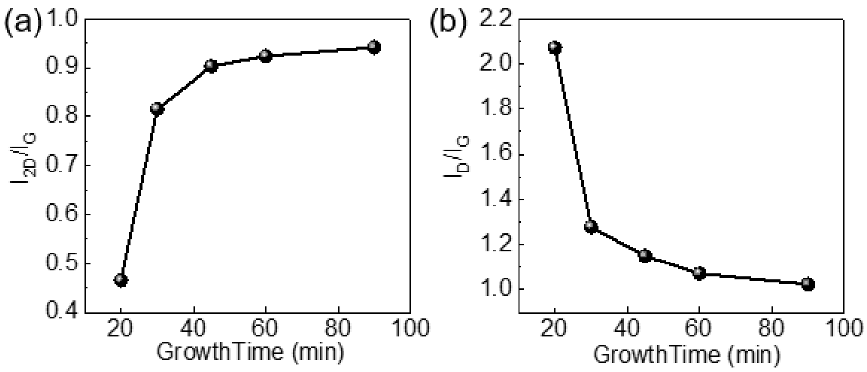

Figure S4. (a,b) I_{2D}/I_G and I_D/I_G ratio of different growth time.

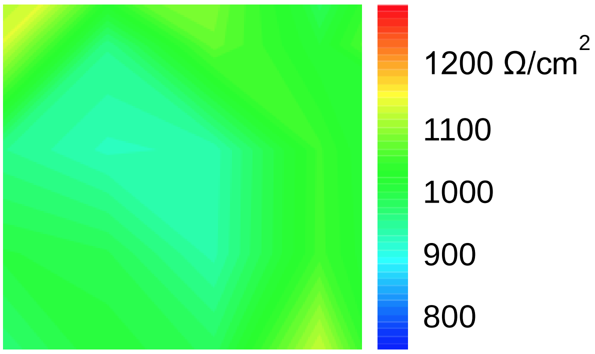

Figure S5. Sheet resistance mapping of sample 45# indicates the excellent uniformity.

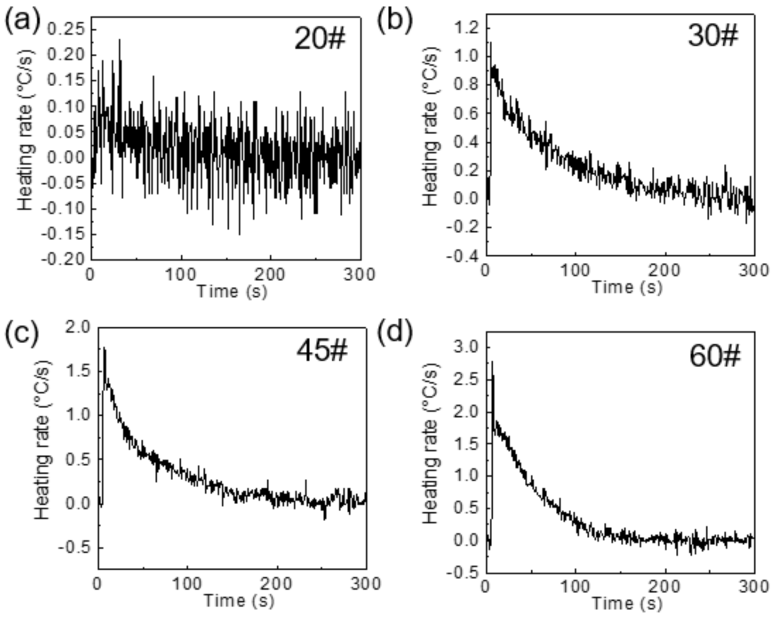

Figure S6. Heating rates of (**a**) 20#, (**b**) 30#, (**c**) 45#, (**d**) 60# at 60 V.

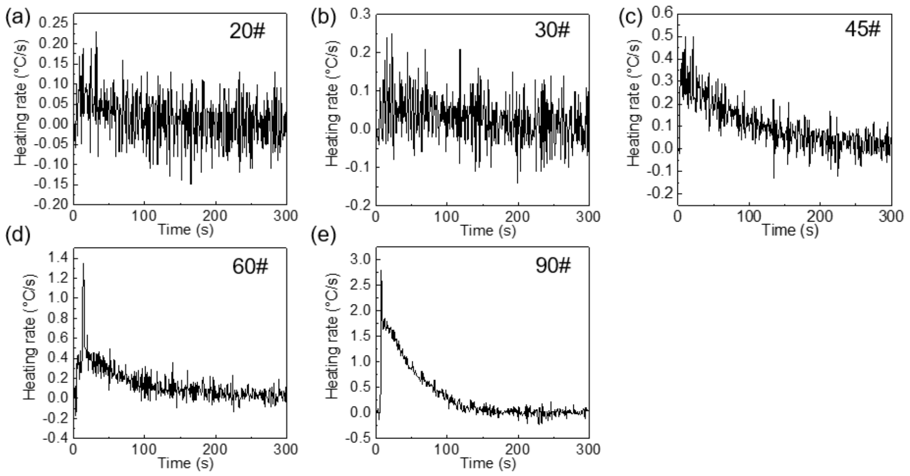

Figure S7. Heating rates of (**a**) 20#, (**b**) 30#, (**c**) 45#, (**d**) 60#, (**e**) 90# at 40 V.

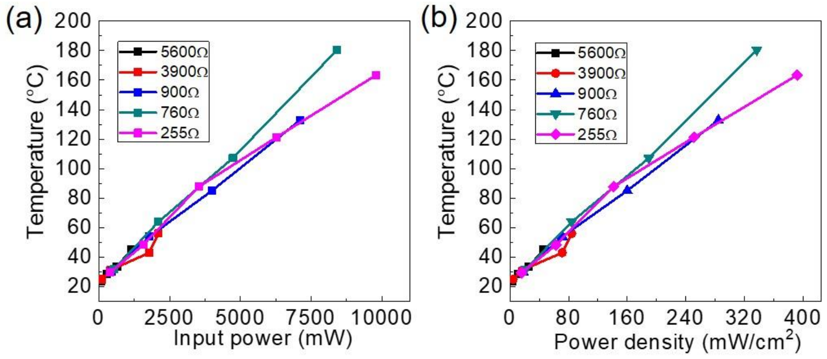

Figure 8. (**a**) steady-state temperatures vs input power; (**b**) steady-state temperature vs power density.

Table S2. Heaters fabricated by carbon-based films and their performances.

Method	Sheet resistance (Ω/□)	*T* max (°C)	Max. heating rate (°C/s)	Ref
Transfer of membrane filtered CNTs onto glass or PET	230–3500	95	1.3	[2]
Transfer of CVD graphene onto glass or PET by etching away the substrate	750	80	0.7	[3]
Roll-to-roll transfer of CVD	66–403	140	0.4	[4]
Roll-to-roll transfer of Au-doped CVD graphene onto PET	43	100	0.8	[5]
This work	255–5600	180	2.8	—

Table S2 summarizes the performances of electrothermal heaters based on reported results. Under the same power density, the max heating rate achieved in this work is comparable, or even larger than that reported previously.

References

1. Y.H. Yoon, J.W. Song, D. Kim, J. Kim, J.K. Park, S.K. Oh and C. S. Han, *Adv. Mater.* **2007**, *19*, 4284–4287.
2. B. J. Lee and G. H. Jeong, *Curr. Appl. Phys.* **2012**, *12*, S113–S117.
3. J. J. Bae, S.C. Lim, G.H. Han, Y.W. Jo, D.L. Doung, E.S. Kim, S.J. Chae, T.Q. Huy, N.V. Luan and Y. H. Lee, *Adv. Funct. Mater.* **2012**, *22*, 4819–4826.
4. J. Kang, H. Kim, K.S. Kim, S.K. Lee, S. Bae, J.H. Ahn, Y.J. Kim, J.B. Choi and B. H. Hong, *Nano Lett.* **2011**, *11*, 5154–5158.
